# Characterizing Initial Cervical Spine and Neurovascular Findings in 84 Consecutive Patients with Hypermobile Ehlers–Danlos Syndrome: A Retrospective Study

**DOI:** 10.3390/jcm15062212

**Published:** 2026-03-14

**Authors:** Ross A. Hauser, Morgan Griffiths, Ashley Watterson, Danielle Matias, Benjamin R. Rawlings

**Affiliations:** 1 Caring Medical Florida, 9738 Commerce Center Court, Fort Myers, FL 33908, USA; rhprolobook@caringmedical.com (R.A.H.); steilend@caringmedical.com (D.M.); 2 Independent Researcher, 9738 Commerce Center Court, Fort Myers, FL 33908, USA; morgriffiths@gmail.com; 3 Division of Behavioral and Organizational Sciences, Claremont Graduate University, 150 East 10th Street, Claremont, CA 91711, USA; ashley.watterson@cgu.edu

**Keywords:** hypermobile Ehlers–Danlos syndrome, cervical instability, cervical lordosis, forward head, internal jugular vein, vagus nerve, autonomic nervous system

## Abstract

**Background**: Hypermobile Ehlers–Danlos syndrome (hEDS) can present as a complex interplay of widespread symptomatology and multisystem involvement, posing diagnostic and treatment challenges. Objective characterization of cervical spine and neurovascular findings in hEDS has been limited. Previous studies have emphasized upper cervical spine complications in hEDS, yet the relevance and mechanisms underlying associated symptomatology have not been elucidated. This study examined objective test findings in patients with hEDS at an outpatient neck clinic to explore cervical spine and neurovascular pathology that could contribute to further understanding the clinical profile of a subset of patients with hEDS. **Methods**: This single-center, retrospective observational study included patients with hEDS aged 20–50 years from 1 January 2022–31 December 2024, at an outpatient neck center. It excluded previous neck surgery, traumatic events, or related injury. Demographic, clinical, and diagnostic data were collected through a retrospective chart review, including measurements from standard clinical diagnostic protocols: digital motion X-ray (videofluoroscopy), cone beam CT, Doppler ultrasound, and tonometry. **Results**: More than 71% of patients reported ≥29 symptoms. Nearly all patients exhibited co-occurring forward head, decreased depth of curve, ligamentous cervical instability, and decreased internal jugular vein (IJV) and vagus nerve cross-sectional area (CSA). Vagus nerve CSA was found to be significantly smaller than the comparative healthy/normal population. IJV CSA was significantly smaller at C1 than at C4–C5, suggesting evidence of carotid sheath compression at C1. **Conclusions**: This study offers novel evidence that cervical spine pathology, IJV compression, and vagus nerve degeneration are uniformly prevalent in hEDS, which may contribute to, or be an etiological basis for, the multisystem involvement in a subset of patients with this disorder. These findings provide hypothesis-generating data to inform future mechanistic and therapeutic studies, including exploration of new diagnostic and treatment targets.

## 1. Introduction

Ehlers–Danlos syndromes (EDSs) are clinically defined as a genetically heterogeneous group of connective tissue disorders, including 13 variants with 21 distinct gene mutations, defined by the 2017 International Classification, and primarily affecting collagen structure [1]. EDS is currently estimated to present in 1 in 500 people based on a recent study involving Welsh healthcare records, which is far greater than the previously thought-to-be-rare estimated occurrence of 1 in 5000 (0.2% vs. 0.002%), though the condition may still be underrepresented due to variability in diagnostic criteria combined with the presence of overlapping conditions [2,3,4]. Hypermobile EDS (hEDS) is characterized by generalized joint hypermobility, musculoskeletal pain, ligamentous joint instability, and a plethora of systemic manifestations often affecting quality of life, the most recognizable clinical signs including musculoskeletal pain, joint hypermobility, skin hyperextensibility, and tissue fragility [5,6]. Hypermobile Ehlers–Danlos syndrome accounts for more than 90% of all EDS cases, with females accounting for 70–90% of hEDS, and as of yet, no definitive genetic mutation has been found, and the cause for the widespread symptomatology is poorly understood [7,8].

Patients with hEDS were selected for this study because their generalized connective tissue laxity, a defining feature of the disorder, may predispose them to cervical spine instability. Previous studies have emphasized upper cervical spine problems in hEDS, ligamentous cervical instability being a frequently considered and potentially disabling complication, yet it has not been consistently examined as a potential structural framework contributing to the broader symptom profile in this population [9,10]. Exploring neurovascular and autonomic dysfunction potentially associated with cervical spine instability in patients with hEDS could lead to characterization of modifiable pathophysiological mechanisms. This characterization could provide a framework for more targeted and mechanistic interventions beyond symptom management and guide individualized therapeutic approaches.

**Figure 1:**
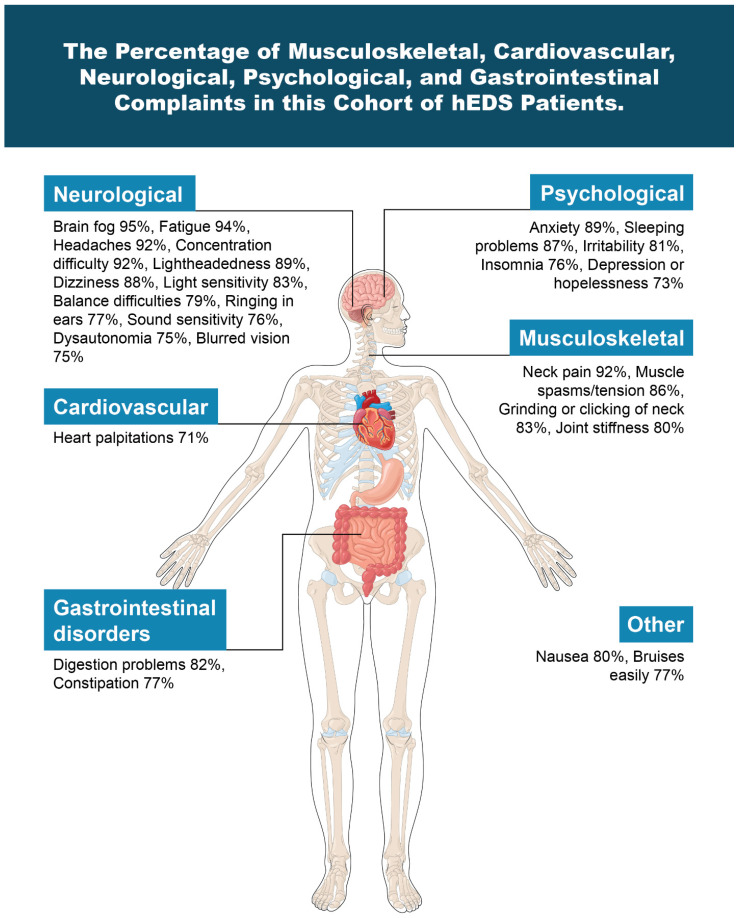
The percentage of musculoskeletal, cardiovascular, neurological, psychological, and gastrointestinal complaints in this cohort of hEDS patients. Figure copyright belongs to authors.

Hypermobile Ehlers–Danlos syndrome patients often experience a wide range of chronic and disabling symptoms, including not only musculoskeletal complaints, but also cardiovascular, neurological, gastrointestinal, and autonomic dysfunctions at a higher rate than the general population, all categories which were highly prevalent in this study [11] (Figure 1). Determining exact frequencies within the general population is inherently difficult for categories of this breadth; however, many investigations have examined them through small-scale comparisons [12,13,14,15]. The exact etiology of the myriad symptoms that patients with hEDS suffer from continues to be a conundrum.

Chronic pain is often the most prevalent symptom, though there are currently no established treatment guidelines for managing or resolving the pain in hEDS patients [16]. Up to 56% of patients with hEDS receive a misdiagnosis, and the complexity of the condition often results in a delayed diagnosis of up to 28 years [17]. Studies have also found that symptom severity progresses over time, and progression is heterogeneous, multisystemic, and unpredictable, even in children [18,19,20]. In a recent cross-sectional, anonymous online survey involving patients with hEDS, participants reported 24 comorbid diagnoses on average, with diagnostic delays averaging over 20 years [3]. Chronic pain was most frequently the most severe symptom, with neck pain being the most frequent site [3].

The chronic disabling and often progressive symptoms of hEDS coupled with treatment uncertainties cause psychological distress and tremendous adverse mental health effects, in addition to the physical symptoms [21,22]. The variability in the range and severity of symptoms, and a lack of uniformity in the etiology of these symptoms, makes treatment plans difficult and often suboptimal, which emphasizes the relevance of investigating potentially treatable underlying mechanisms [23]. Despite emerging evidence of dysautonomia, altered cerebrovascular control, and neuropathic features present in hEDS, multisystem abnormalities have rarely been considered together in a unified framework that could explain the widespread symptom patterns, making it difficult to infer integrated mechanisms that could link neuromuscular and autonomic manifestations of this condition [24,25]. Consequentially, there is limited high-quality evidence of treatment approaches for the widespread severely disabling symptoms, and current management remains symptom-based rather than being guided by pathophysiological pathways [8,26,27].

The aim of this study was to further understand the clinical profile of patients with hEDS at an outpatient neck center by exploring potential cervical spine pathology, including ligamentous cervical instability, and identifying pathophysiological patterns prevalent in the cohort which could elucidate mechanisms to potentially help explain the widespread symptomology and multi-system involvement in this condition. Current hEDS treatment is aimed at pain and symptom management and improving quality of life, focused on pharmaceuticals, lifestyle modifications, bracing, physical therapy, education, and psychological intervention, often involving multiple different teams of specialists, all of which pose significant diagnostic and treatment challenges [28]. To our knowledge, systematic evaluation of cervical spine instability and adjacent neurovascular findings in patients with hEDS has not been studied. This study adds to the current literature by identifying consistent cervical spine and neurovascular findings in a cohort of patients, providing preliminary data that may inform future investigations into potential structural and autonomic contributors to symptom burden in hEDS, and possibly leading to new diagnostic and treatment targets.

## 2. Methods

### 2.1. Study Population

In this retrospective, descriptive, observational study, 84 consecutive patients with hEDS were identified from a clinical database review of patients reporting to The Hauser Neck Center (an outpatient neck clinic in Fort Myers, Florida, USA) from 1 January 2022–31 December 2024. The clinic has an international referral base, and patients come primarily to see if they have a structural neck issue, specifically ligamentous cervical spine etiology, as a potential cause or contributor to their varied disabling symptoms. All patients aged 20–50 with an hEDS diagnosis who met the 2017 International Classification criteria were included. We excluded patients who had a history of neck surgery, traumatic events, or related injuries. The 84 patients included represent all consecutive individuals meeting eligibility criteria during the study period. They were not preselected based on imaging findings or symptom severity. Clinical care was delivered according to standard protocols, and the retrospective analysis was conducted subsequently. This study was designed as a descriptive, cross-sectional characterization of initial clinical test findings. No additional subgroup analyses were performed.

**Figure 2:**
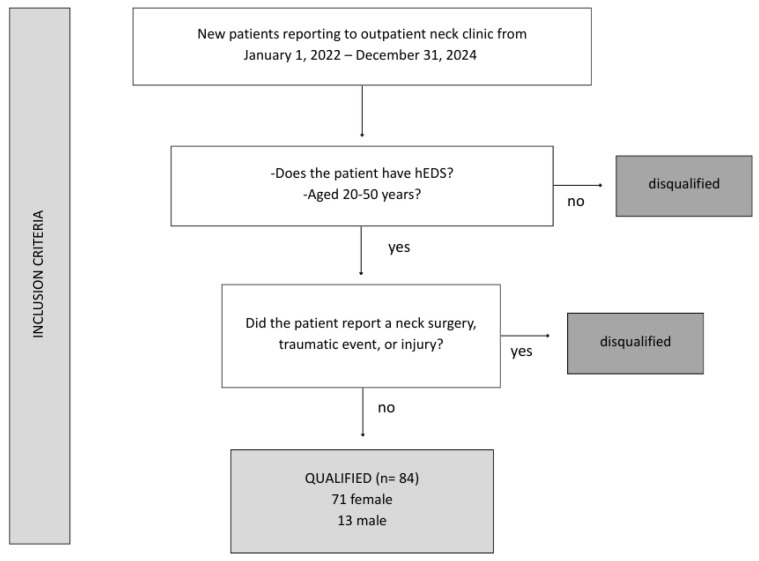
Patient inclusion flow chart. Figure copyright belongs to authors.

The patient group was 15% male and 85% female. Informed consent was obtained from the subjects after an explanation of the nature and possible consequences, and the study was approved by the WIRB-Copernicus Group (WCG) Institutional Review Board (Study #20252006) (Figure 2).

**Figure 3:**
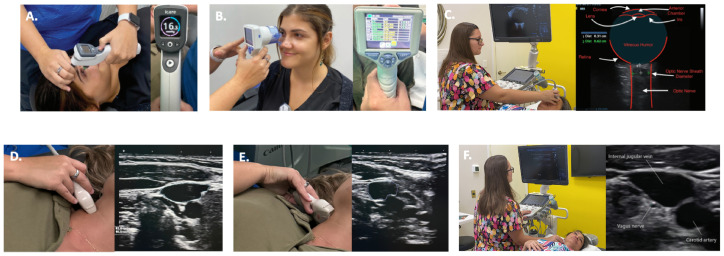
Neck vitals analysis. (**A**) Tonometry. (**B**) Pupillary light reflex. (**C**) Optic nerve sheath diameter. (**D**) Internal jugular vein (IJV) cross-sectional area (CSA) at C4–C5. (**E**) IJV CSA at C1. (**F**) Vagus nerve CSA. Figure copyright belongs to authors. (Reproduced from Ref. [29]).

Demographics, symptoms, and clinical test results were collected by chart review, including age, gender, upright digital motion X-ray (DMX, videofluoroscopy), and upright cone beam CT (CBCT) of the cervical spine, pupillometry, tonometry, and ultrasound of the carotid sheath (internal jugular veins and vagus nerves) and the eye (optic nerve) (Figure 3). All diagnostic procedures were performed by a radiology technologist or medical ultrasonographer for clinical (not research) purposes. All in-clinic imaging and testing were performed following standardized clinical protocol, minimizing potential bias. Formal research blinding was not implemented due to the retrospective study design. Selection bias is inherent to this retrospective single-center study design, potentially limiting the generalization of the findings, although it allows for detailed characterization of patients with hEDS seeking care at a self-referred neck clinic.

This study included only symptom frequencies and data collected at initial intake. Symptoms were included in the analysis if >70% of patients reported them, resulting in 29/81 symptoms included. Follow-up data were not included. Symptoms documented at initial intake are reported as frequencies, with the number and percentage of participants experiencing each. The objective test results (quantitative variables) were analyzed as continuous variables. Descriptive statistics included means and standard deviations. No confounding variables were identified or adjusted for in this analysis, which already excluded previous injuries and traumatic events. This study did not include an analysis of effect modifiers, though modifications such as head position and movement may be considered in future investigations. This retrospective study did not include a control group. Where applicable, measurements were compared with published normative reference values.

**Figure 4:**
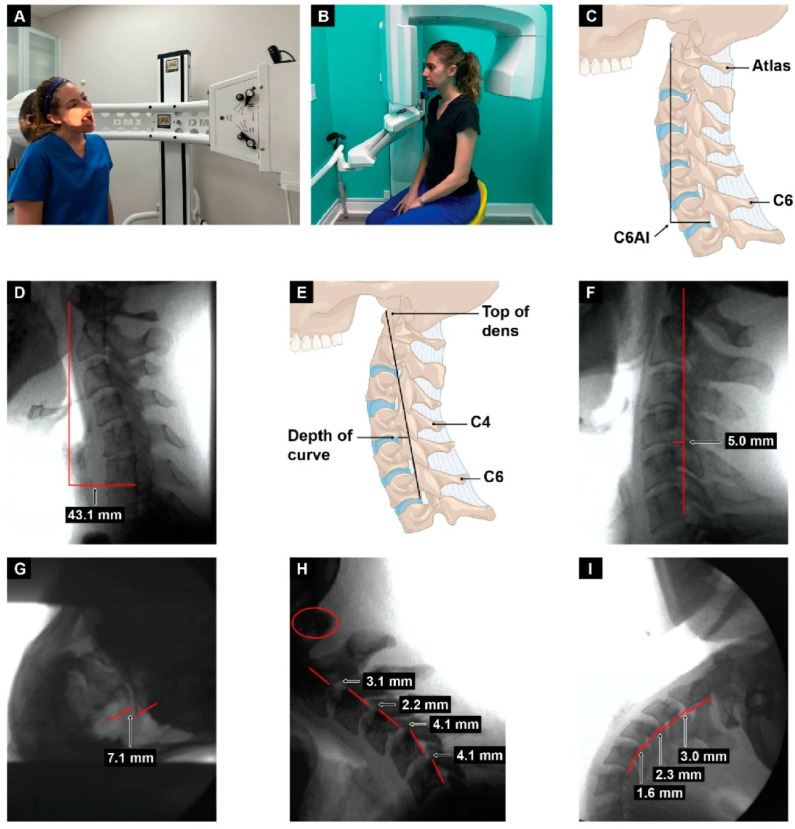
Upright digital motion (fluoroscopic) X-ray (DMX) and cone beam computed tomography (CBCT) scan with structural measurements. (**A**) DMX positioning for open mouth lateral flexion. (**B**) CBCT setup. (**C**) Forward head (C6AI*) illustration. (**D**) C6AI measurement. (**E**) Depth of curve** illustration. (**F**) Depth of curve using DMX. (**G**) C1–C2 instability measurement. (**H**) Flexion, lower cervical instability. (**I**) Extension, lower cervical instability. * C6AI = horizontal distance in the sagittal plane of the posterior inferior C6 vertebra to anterior atlas (optimal is <10 mm). ** Depth of curve = horizontal distance in the sagittal plane from posterior inferior C4 vertebra to line drawn from posterior inferior C6 vertebra to top of dens (optimal is 7–17 mm). Figure copyright belongs to authors. (Reproduced from Ref. [29]).

Measurements used to evaluate for ligamentous instability in the lower cervical spine (C2–C6) in flexion and extension, and the upper cervical spine (C1–C2) in lateral flexion with open mouth views, were obtained following DMX. DMX (videofluoroscopy) is a reliable method with a high degree of accuracy when assessing for cervical spine instability [30]. All patients had lateral X-ray imaging during maximum protraction to ensure the anterior atlantodens intervals were within normal range (<3 mm) [31]. Measurements to evaluate the state of cervical lordosis—depth of curve and forward head placement in relation to the lower cervical spine (C6-atlas interval)—were obtained following CBCT (Figure 4 and Table 1).

Neck vitals testing provided measurements of pupil diameters to assess for dilation and pupillary light response, optic nerve sheath diameters (ONSDs) for increased fluid around the optic nerve, internal jugular vein cross-sectional areas for structural compression and/or venous outflow obstruction from the brain, vagus nerve cross-sectional areas for degeneration, and intraocular pressure for increased pressure within the eye (Table 2).

### 2.2. Statistical Analysis

Data was analyzed using RStudio version 2024.04.2+764. Continuous variables were summarized using means and standard deviations, and categorical variables (e.g., number of symptoms) were summarized using counts and valid percentages. Statistical significance was defined as *p* < 0.05 for all tests.

We retained the full analytic sample (N = 84) and chose not to remove univariate outliers, as all values were clinically valid, accurately recorded, and confirmed through multiple quality checks. This decision was made to preserve the full range of clinical variations present in the sample, which reflects real-world patient presentations. Although some values were extreme, they did not result from data entry errors and were considered meaningful for analysis.

Missing data were handled using a pairwise deletion approach, meaning that each statistical test included all available cases for the variables involved. This approach preserved the maximum number of observations per analysis, and the sample size used in each test is reported accordingly.

Assumptions for parametric testing were evaluated. Skewness and kurtosis values for continuous variables fell within acceptable ranges (±3 and ±10, respectively). Normality of difference scores for paired comparisons were confirmed using Shapiro–Wilk tests (*p* > 0.05), and linearity of continuous variable relationships was verified using scatter plots. While certain variables showed non-normal distributions, Pearson correlations were appropriate due to the observed linearity in relationships and the robustness of this method in moderate-to-large samples.

To compare IJV CSAs across positions (C1 supine, C4–C5 supine, and C1 supine on Denneroll^®^, made in Wheeler Heights, NSW, Australia), paired *t*-tests were conducted. Because assumptions of normality for the paired difference scores were met, *t*-tests were the primary method of comparison. However, Wilcoxon signed-rank tests were also conducted as a robustness check. In all cases, parametric and non-parametric results were consistent, reinforcing the stability of the findings.

No covariates were included in the analysis, and subgroup or interaction effects were not examined due to the exploratory nature of the study. All analyses were conducted using raw, complete-case data without sampling weights or design-based corrections.

## 3. Results

Of the 84 patients included, 71 (85%) were female, 13 (15%) were male, average age 35 years, ranging from 20 to 50 years. Symptoms analysis showed that the top five reported symptoms at initial presentation were brain fog (95%), fatigue (94%), headaches (93%), concentration difficulty (92%), and neck pain (92%). A total of 83.1% of the patients self-reported more than 20 symptoms during their initial intake (Table 3).

Analysis of clinical radiographic measurements gathered by retrospective chart review revealed 98.8% to have an increased (>10 mm) [32] C6-atlas interval (radiographic measurement of structural forward shift in the atlas in relation to lower cervical spine), 96.4% to have ligamentous cervical instability in lateral flexion at C1–C2 (>2 mm) [33,34], and 94.9% to have decreased depth of cervical lordotic curve (normal is 7–10 mm) [35,36].

Analysis of clinical objective neck vitals gathered from retrospective chart review showed elevated (>6.1 mm) [37,38] ONSD in 87.9% and 80.7% of participants on the left and right respectively, and slightly elevated pupil diameters (>4 mm) [39,40] in 95.2% in both left and right eyes. Vagus nerves were shown to be decreased (normal being >2.1 mm on right, >1.9 mm on left) [41,42] in 98.8% and 91.7%, respectively. The IJV CSA at C1 in the supine position was below normal limits (<90 mm) [43,44,45] in 91.7% and 95.2% on the left and right, respectively (Table 4).

We also analyzed the mean and standard deviation of each cervical structural measurement and neck vitals test results, which revealed that many values fell outside the normal range, on average, within this patient cohort. The mean C6-atlas interval was 39.12 mm, which objectively measures the structural forward position of the atlas in comparison to the lower cervical spine, providing a radiographic measurement for evaluating forward head posture. Depth of curve mean was 1.58 mm, which objectifies loss of cervical lordosis. The mean ligamentous cervical instability at C1–C2 in lateral flexion (total, left and right) was 7.84 mm. Flexion and extension instability means were 5.29 mm and 4.41 mm, respectively (total from C2–C6, normal <4 mm). Flexion and extension instability (retrolisthesis anterolisthesis) are measured in maximum flexion and maximum extension. IJV CSAs in the supine position were decreased in all locations, the means being 40.97 mm^2^, 39.94 mm^2^, 67.91 mm^2^, and 71.48 mm^2^ at C1 left, C1 right, C4–C5 left, and C4–C5 right, respectively. Mean optic nerve sheath diameter was 7.28 mm and 7.17 mm on the left and right, respectively. Mean vagus nerve cross-sectional area was 1.34 mm^2^ and 1.22 mm^2^ on the left and right, respectively (Table 5).

### 3.1. Comparisons

#### 3.1.1. Vagus Nerve Cross-Sectional Area in hEDS vs. Control

An independent samples *t*-test was conducted to compare total vagus nerve CSA between patients with hEDS and healthy controls reported in a prior study (Bedewi, et al., 2023 [42]). Hypermobile Ehlers–Danlos syndrome patients had significantly smaller vagus nerve CSA (M = 2.55 mm^2^, SD = 0.59) than controls (M = 4.00 mm^2^, SD = 0.78), *t*(125) = −11.72, *p* < 0.001. The standardized effect size was Cohen’s *d* = −2.20, indicating a very large difference between groups. According to conventional benchmarks, values of *d* ≥ 0.80 are considered large, thus this result reflects a substantial reduction in vagus nerve size among this cohort of patients with hEDS compared to healthy individuals.

#### 3.1.2. IJV CSA C1 Supine vs. IJV CSA Denneroll^®^

**Figure 5:**
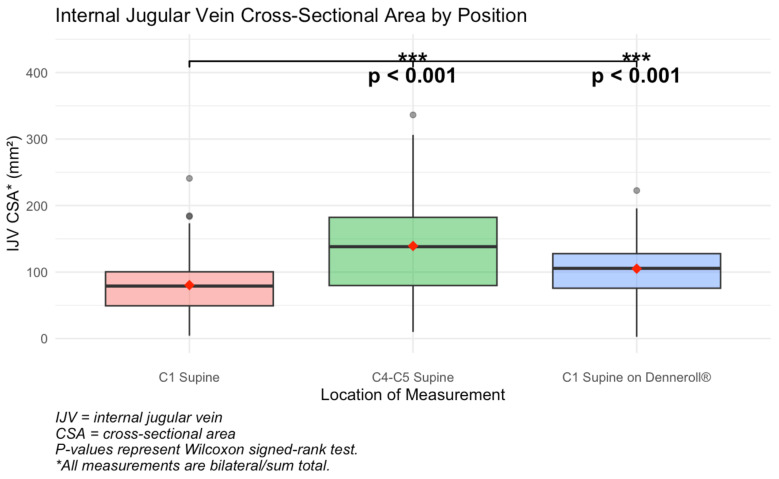
Comparison of IJV C1 supine to IJV C4–C5 and C1 supine on Denneroll^®^ showing that IJV CSA was significantly smaller at C1, and increased with use of cervical orthotic device, the Denneroll^®^, which is used to enhance the cervical curve. These comparisons suggest IJV CSA at C1 increases with improvement of cervical lordosis. Figure copyright belongs to authors.

A Shapiro–Wilk test confirmed that the different scores between IJV CSA at C1 supine and C1 on Denneroll^®^ were normally distributed (*W* = 0.98, *p* = 0.358), meeting assumptions for parametric analysis. A paired *t*-test revealed a statistically significant difference between the two positions, *t*(79) = −4.58, *p* < 0.001. The mean difference was −23.27 mm^2^, with a 95% confidence interval ranging from −33.38 to −13.16. These results indicate that, on average, IJV CSA at C1 while lying on the Denneroll^®^ is significantly higher than IJV CSA at C1 supine. To confirm the robustness of this finding, a Wilcoxon signed-rank test was also conducted and yielded a consistent result, *V* = 719.5, *p* < 0.001 (Figure 5).

#### 3.1.3. IJV CSA C1 Supine vs. IJV CSA C4–C5 Supine

Similarly, the Shapiro–Wilk test indicated that the difference scores between IJV CSA at C1 and C4–C5 supine were normally distributed (*W* = 0.981, *p* = 0.240). A paired *t*-test showed a significant difference, *t*(83) = −8.83, *p* < 0.001. The mean difference was −58.82 mm^2^, with a 95% confidence interval of −72.06 to −45.57, suggesting that IJV CSA at C1 supine is significantly lower than at C4–C5 supine. The Wilcoxon signed-rank test confirmed this result, *V* = 275, *p* < 0.001, reinforcing the robustness of the finding across both mean and median comparisons (Figure 5).

## 4. Discussion

This study found that more than 90% of patients demonstrated abnormal findings of LCI C1–C2 lateral flexion, C6AI, and depth of curve, more than 95% had evidence of bilateral IJV stenosis and elevated ONSD, and 100% had abnormally small vagus nerve CSA, indicative of nerve degeneration/atrophy. The high prevalence and overlap of these structural abnormalities and test findings suggest these results are unlikely to be coincidental and raise the possibility that these findings may be mechanistically linked. We hypothesize that the findings likely represent cervical spine involvement contributing to the neurologic and autonomic features and systemic symptoms commonly found in hEDS.

**Figure 6:**
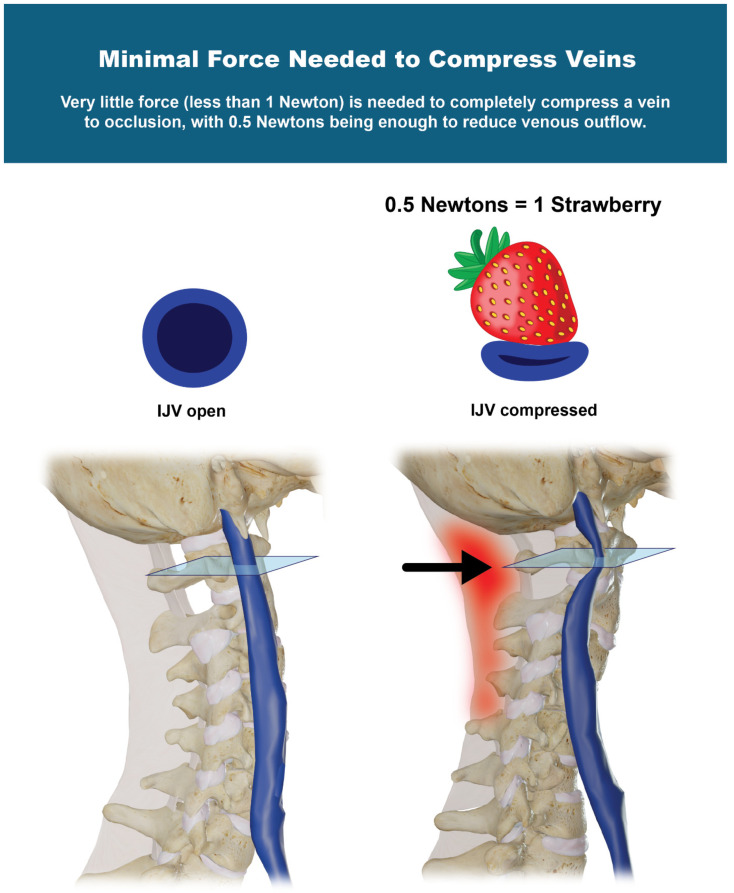
Very little force (less than 1 Newton) is needed to completely compress a vein to occlusion, with 0.5 Newtons being enough to reduce venous outflow. As the posterior ligaments of the cervical spine become damaged (red), the atlas shifts forward in 3-D space (arrow), causing compression of the internal jugular vein. Figure copyright belongs to authors.

Very little force (less than 1 Newton) is needed to completely compress a vein to occlusion, with 0.5 Newtons being enough to reduce venous outflow, equivalent to the very little force needed to click a pen [46,47] (Figure 6). As little as 0.5 Newtons has also been shown to cause compression injury on nerves, with effects of reducing conduction velocity, decreasing microvascular flow, and altering myelin structure from mechanical shear force [48,49].

**Figure 7:**
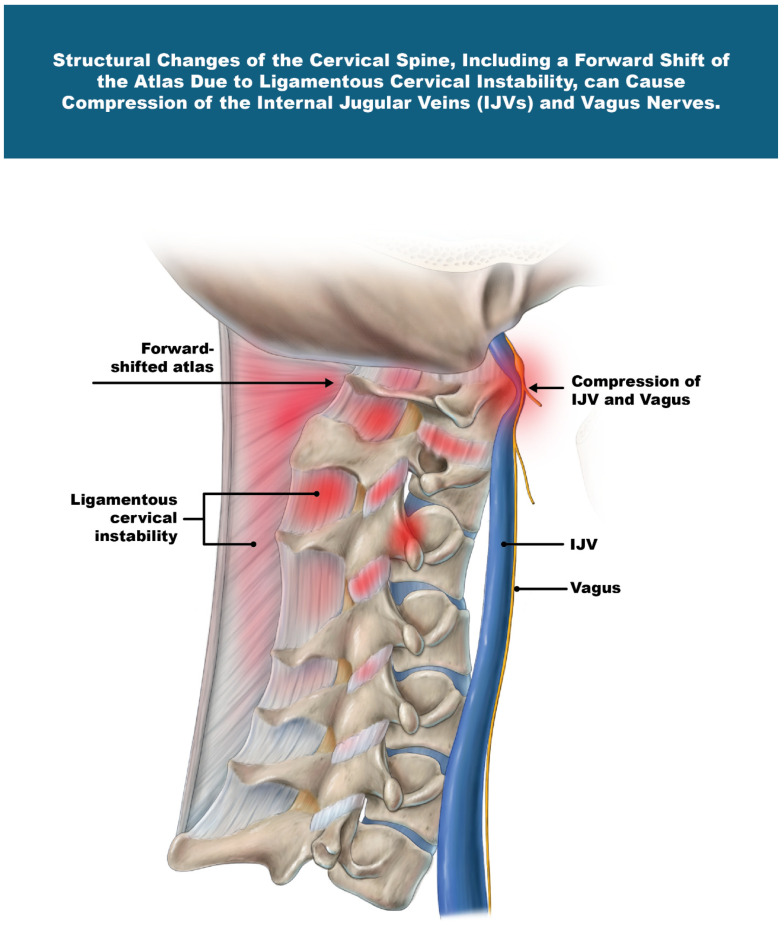
Structural changes in the cervical spine, including a forward shift in the atlas due to ligamentous cervical instability, can cause compression of the internal jugular veins (IJVs) and vagus nerves. Figure copyright belongs to authors.

The IJVs and vagus nerves run within the carotid sheath and do not have cartilage protection, making them vulnerable to compression and injury. We postulate that the cervical structural abnormalities (LCI, depth of curve, and C6AI) documented in this study cause compression of the carotid sheath, primarily due to a forward shift in the atlas, documented as decreased CSA of the vagus nerves and IJVs. (Figure 7). Collectively, these findings suggest that cervical spine structural abnormalities may underly this cohort’s multisystem symptomatology, pointing toward potential involvement of impaired venous outflow and altered autonomic regulation due to IJV compression and vagus nerve degeneration, respectively.

A comparison of IJV CSAs at C1 and C4–C5 showed that supine IJV CSA C1 total (IJV bilateral totals at the atlas) was significantly smaller than in the mid-cervical region (C4–C5), with an average difference of 58.82 mm^2^ (*p* < 0.001), suggesting IJV compression at C1. This comparison shows that IJV compression could be missed if IJV CSA is only evaluated at the mid-neck region, where ultrasound evaluation of the IJV is most often utilized [50].

The significantly smaller average vagus nerve CSA (bilateral total of 2.55 mm^2^) in this cohort compared to an asymptomatic, healthy, demographically similar population’s average vagus nerve CSA (4.0 mm^2^) indicates the presence of vagus nerve degeneration (*p* < 0.001) [42].

**Figure 8:**
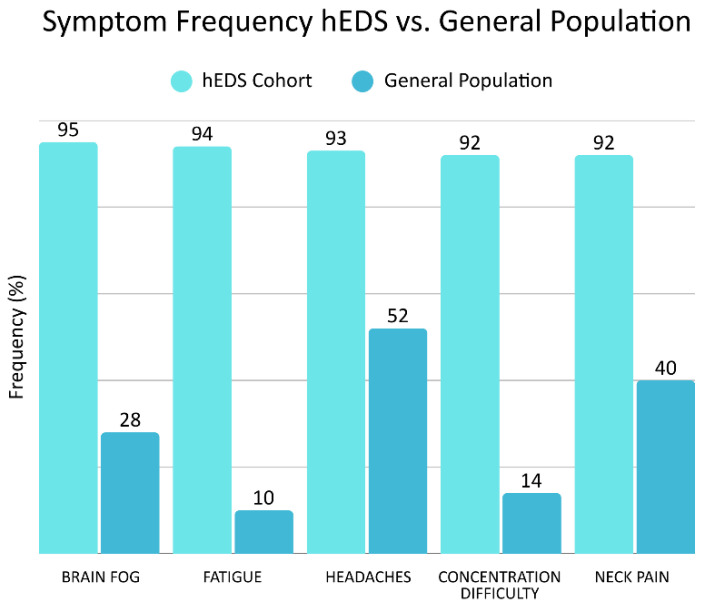
Symptom frequencies in this hEDS cohort compared to general population. Figure copyright belongs to authors.

More than 83% of our cohort reported more than 20 symptoms on initial presentation, the top five being brain fog, fatigue, headaches, concentration difficulty, and neck pain. Chronic disabling symptoms involving the musculoskeletal, ocular, cardiovascular, gastrointestinal, and other body systems and organs are many times more prevalent in hEDS vs. the general population, as seen in our cohort: brain fog (95% vs. 28%), fatigue (94% vs. 10%), headaches (93% vs. 52%), concentration difficulty (92% vs. 14%), and neck pain (92% vs. 30–50%) [11,51,52,53,54,55,56,57] (Figure 8). The symptoms reported in this study (and others) are frequently concurrent comorbidities in hEDS patients, and many co-occurring conditions act as disabling conditions on their own, which adds to treatment difficulties and emphasizes the systemic burden of hEDS [2].

**Figure 9:**
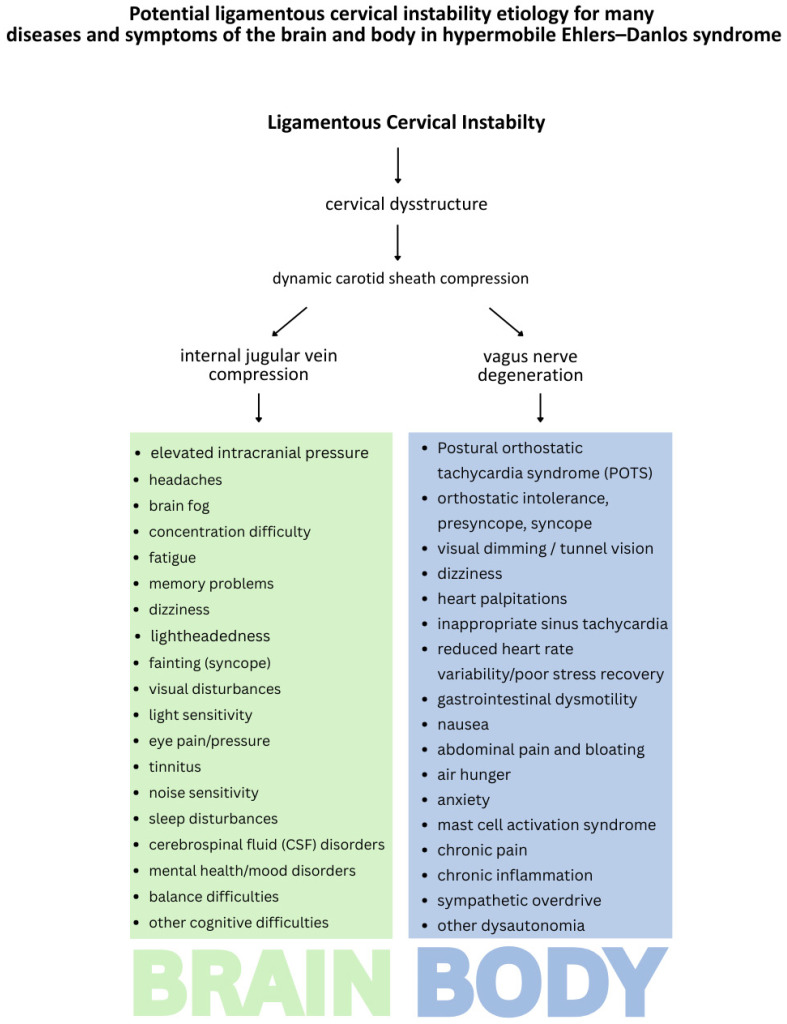
Potential ligamentous cervical instability etiology for many diseases and symptoms of the brain and body in hypermobile Ehlers–Danlos syndrome [32,58,59,60,61,62,63,64,65]. Figure copyright belongs to authors.

We suspect the documented LCI, breakdown of the cervical curve (decreased depth of curve), and forward head posture (measured as C6AI) contributes to a forward shift in the atlas in 3-D space, putting stretch and compression on the soft tissues and neurovascular contents of the neck, including the carotid sheath, containing the IJVs and vagus nerves. The right and left carotid sheaths contain the internal carotid artery, internal jugular vein, vagus nerve, glossopharyngeal nerve, and spinal accessory nerves. This dynamic carotid sheath compression, which refers to carotid sheath compression with certain head and neck positions that compromise the structures within the carotid sheath, could be one explanation for many symptoms of hEDS, potentially accounting for various brain and body diseases and symptoms, including many seen in this patient cohort: headaches, brain fog, concentration difficulty, vision issues, lightheadedness, fatigue, memory problems, tinnitus, sleep disturbances, anxiety, depression, heart palpitations, gastrointestinal problems, chronic pain, and more [32,58,59,60,61,62,63,64,65] (Figure 9).

Various explanations have been put forth regarding the diverse symptomatology in hEDS, including genetic and nongenetic factors, recent findings connecting kallikrein genes, faulty collagen making the ligaments more stretchy, neurovascular dysfunction, increased vascular endothelial permeability, gastrointestinal hypermobility, neuromuscular proprioceptive defects, autonomic nervous system dysfunction (dysautonomia), histamine dysregulation, soft tissue lesions (e.g., bursitis, tendonitis, synovitis, tenosynovitis, and fasciitis), fascial, vascular, and inflammation [17,66,67,68,69]. We propose that dynamic carotid sheath compression could either be a primary structural etiology or act as a contributing factor alongside the other mechanisms outlined above. Further studies are needed to determine clinical relevance of cervical dysstructure, IJV compression, and vagus nerve degeneration in patients with hEDS.

### 4.1. Ligamentous Cervical Instability

**Figure 10:**
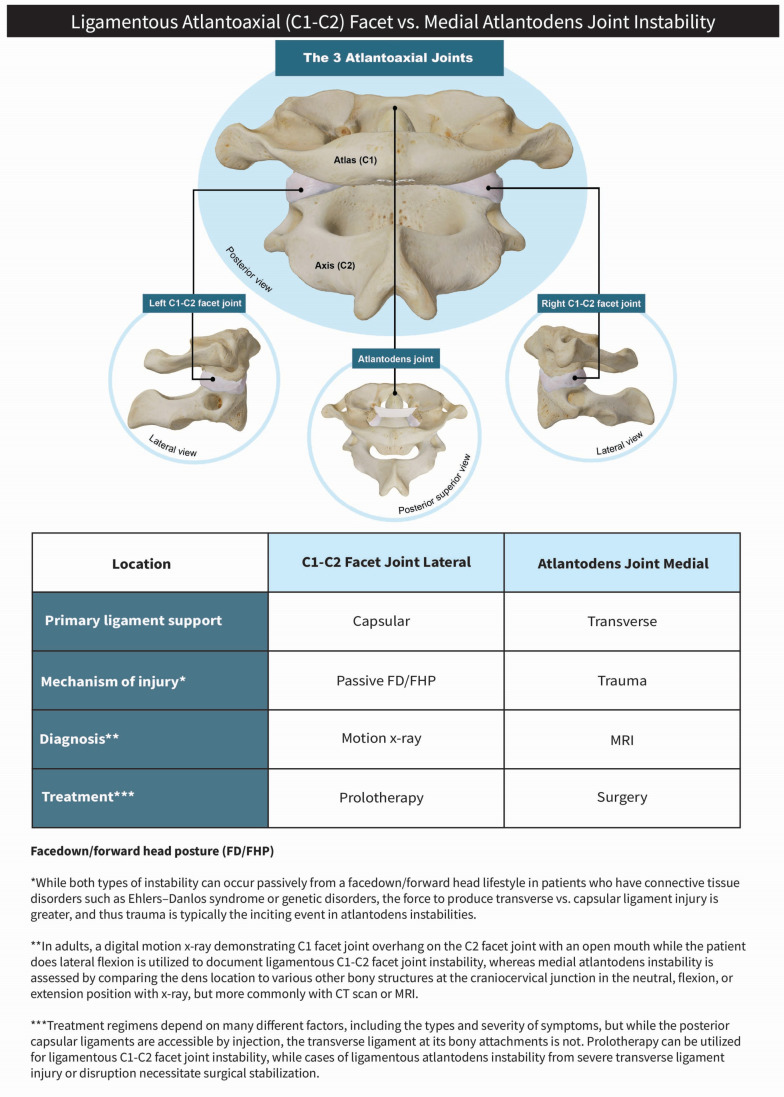
Ligamentous atlantoaxial (C1–C2) facet vs. medial atlantodens joint instability. Figure copyright belongs to authors.

This is the first study that documents ligamentous facet joint cervical instability at C1–C2 (LCI C1–C2) in a cohort of hEDS patients. Recently, spinal deformities in EDS are being addressed more prominently, but without specific details on hEDS or facet joint instability [70]. The previously documented ligamentous cervical instabilities in EDS patients are of the atlantodens, which often require surgical intervention and differ from our cohort, where instability of the facet joints was documented [71]. In the medical literature, atlantoaxial instability usually refers to ligamentous atlantodens (or central or medial atlantoaxial) instability, describing the relationship between the atlas, dens, and medial joint location, primarily stabilized by the transverse and alar ligaments [72,73]. In contrast, the atlantoaxial facet joints (left and right) are located laterally and stabilized by the capsular ligaments, and thus the condition is also known as lateral atlantoaxial instability when injured [74,75,76] (Figure 10). The distinction between the two different types of C1–C2 instability is important because atlantoaxial instability is typically a surgical lesion, whereas atlantoaxial facet joint instability can often be treated by conservative measures such as chiropractic care, physiotherapy, and prolotherapy [71,77]. In this cohort, patients were clinically confirmed to *not* have atlantoaxial instability by measuring the atlantodental interval during clinical testing and confirming each patient had less than 3 mm, which is the standard cutoff to be considered abnormal in adults [78].

Diagnostic and management complexities of craniocervical instability in EDS patients have been previously discussed, largely highlighting the under-researched aspects of the conditions, and a need for greater consensus and guidelines to be validated regarding when surgery is necessary and to explore other potential treatment strategies [79]. A high percentage of hEDS patients who undergo spine surgery report complications (42.9% in a cohort of more than 1300 patients) [14].

In our patient population, we performed *dynamic* upper cervical functional fluoroscopy, which demonstrated stable anterior atlantodens joints (<3 mm distance between arch of C1 and dens of C2 on maximum protraction views) but significantly unstable C1–C2 facet joints (>2 mm translation/overhang of C1 on C2 on lateral flexion views) [80,81]. Congenital capsuloligamentous laxity is the primary articular feature of hEDS [82]. Ligamentous upper cervical instability is a common finding in severely symptomatic hEDS patients, already thought to complicate EDS presentation [64,83]. LCI is characterized by vertebral translation > 2 mm in one direction, defined by the degree of overhang by an adjacent vertebra, though this criterion still needs further clinical validation, as some patients can be symptomatic with less translation [30,33,84]. Some centers consider a difference of ≥1 mm between maximum flexion and extension x-rays to be diagnostic [34]. Our patient population had both extension and flexion LCI, demonstrating > 1 mm of difference between vertebrae at multiple levels, as well as C1–C2 facet joint instability. Highly suggestive symptoms of ligamentous upper cervical instability include heavy head/bobblehead, apprehension of neck movement, difficulty swallowing, clicking/grinding in the neck, sensorimotor symptoms such as a pulling sensation of the head/neck, numbness/tingling, vision changes, cognitive changes, drop attacks, and more [85]. Symptoms of ligamentous upper cervical instability may increase with forward head posture, leaning forward, neck motion, neck flexion, or being upright without neck support [85].

The musculoskeletal manifestations of hEDS are widespread, often culminating in a high percentage of patients having chronic pain and neurological symptoms, which was seen in the 92% of this cohort having chronic neck pain, headaches, and concentration difficulty, 95% having brain fog, and more than 85% having dizziness, anxiety, lightheadedness, and much more [86,87]. Emerging evidence shows upper cervical spine morphology to be affected in hEDS patients significantly more often than in controls, joint instability and muscle imbalances being potential contributors to forward (extended) head posture, which may worsen cervical pain and dysfunction [9]. Based on this study, one possible cause of pain could arise from the nociceptors (from the Latin *nocere*, “to harm or hurt”) in the ligaments: 50% of the these sensory neurons are in C1–C3 region, the very area where the most significant ligament instabilities were found in this cohort [88,89].

**Figure 11:**
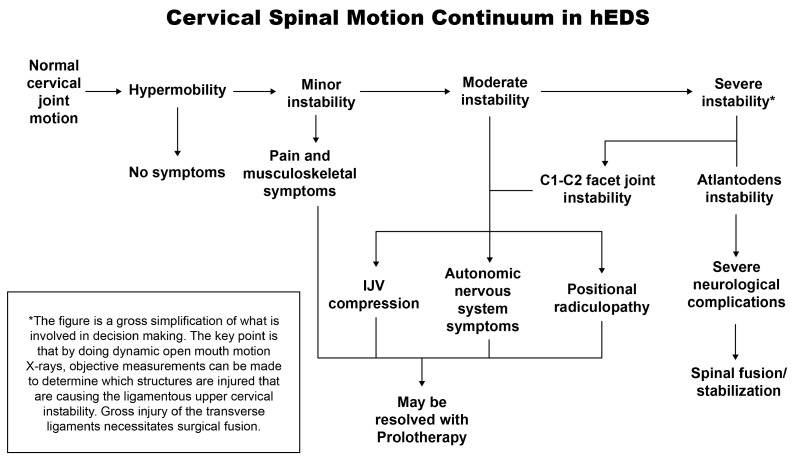
Joint hypermobility may progress into ligamentous cervical instability due to excessive forces on the cervical spine, and ligamentous cervical instability will continue to progress unless the forces from the cervical dysstructure are relieved. Figure copyright belongs to authors.

The combination of the capsuloligamentous issues in hEDS compounded with the multidimensional (flexion, extension, and lateral flexion) ligamentous cervical instabilities found in this cohort could contribute to the development of structural forward head posture (increased C6AI) and loss of cervical lordosis (decreased depth of curve) [90]. As a person tilts their head forward, the forces on the craniocervical junction are amplified, eventually causing long-lasting stretching, assumed to be especially hazardous to joint stability [91]. A 15° tilt, for instance, causes the average head to feel like 27 pounds, and at a 45° tilt, it feels like it weighs around 49 pounds [92]. A forward head posture puts strain on the posterior cervical ligaments, potentially leading to a breakdown of the normal cervical lordotic curve, the cumulative structural changes being termed “cervical dysstructure.” [93]. In theory, then, simple daily facedown/forward head posture, such as looking down at a phone held a lap, is a long-term and repetitive amplified force on the craniocervical junction, which could lead to cumulative structural changes that likely stem from ligament damage. Unless something is done to stop the process of abnormal forces on the cervical spine causing the ligament laxity and the structural changes, cervical dysstructure will likely be progressive, and so could be the symptomatology [91] (Figure 11).

Cervical spine instability in hEDS is already attributed to ligament laxity [94]. A 2023 study observed that patients with radiographically confirmed upper cervical instability (using DMX) and loss of cervical curve after trauma showed improvement in both cervical lordosis and upper cervical instability following a cervical curve correction regimen, as well as statistically significant symptomatic and functional improvement, supporting the link between these two measures and clinical outcomes [95]. Further studies are needed to elucidate the possible interrelationships between ligamentous upper cervical instabilities, increased C6AI (forward head), and decreased depth of curve (loss of lordosis). Additionally, future research should assess the effects of those structural changes on the soft tissues, nerves and vasculature in the neck, and clinical outcomes following cervical structural restoration.

### 4.2. Internal Jugular Vein Compression at C1 Involvement in hEDS Patients

**Figure 12:**
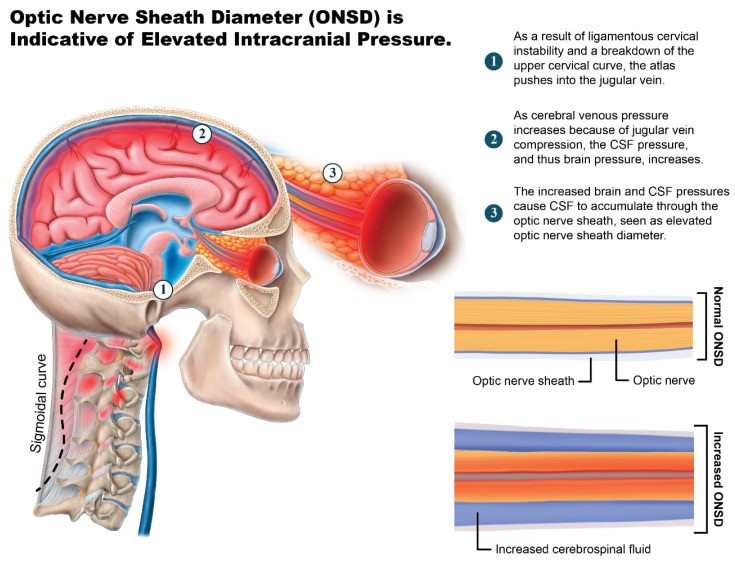
Elevated optic nerve sheath diameter (ONSD) is indicative of elevated intracranial pressure. Figure copyright belongs to authors.

The overwhelming frequency of documented IJV compression at the level of C1 in this patient cohort suggests venous obstruction as a contributing factor to hEDS symptoms. The IJVs span the cervical spine medial to the lateral mass of C1, running along the transverse process of C1, which is where most IJV compression occurs [96]. IJV compression was documented at the level of C1 (atlas) in 96.4% of this cohort, while 92.8% displayed elevated ONSD. Elevated ONSD was not a surprising finding, given the frequency of IJV compression, which is understood to be a mechanism of inhibiting cerebral venous outflow, including cerebrospinal fluid (CSF) drainage [97] (Figure 12). Based on these findings, we suspect venous and CSF drainage to be impaired in this cohort due to the IJV compression from a forward shift in the atlas, given the smaller IJV CSA values at C1 which could cause increased intracranial pressure and resultant increased ONSD. IJV compression is one mechanism which may explain some of the brain-based symptoms experienced by the patients in this study, including headaches, brain fog, concentration difficulty, fatigue, dizziness, blurred vision, depression, and anxiety [98,99,100].

**Figure 13:**
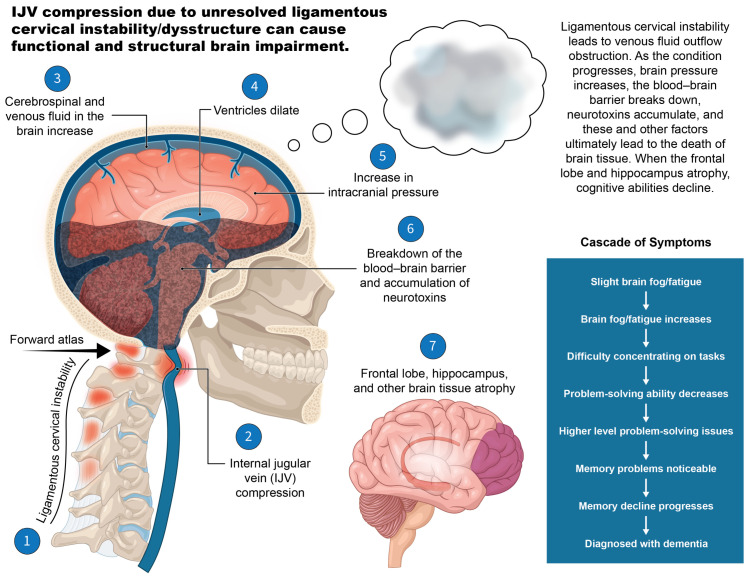
IJV compression due to unresolved ligamentous cervical instability/dysstructure can cause functional and structural brain impairment. Figure copyright belongs to authors.

There are many ways in which IJV compression could cause functional and structural brain impairment, including raising intracranial pressure, decreasing cerebral perfusion, causing change in cerebrospinal fluid dynamics, inciting cerebral microvascular structure impairment, and contributing to a breakdown of the blood–brain barrier, among others [101,102,103] (Figure 13). Structural and functional brain changes are frequently present in hEDS, specifically disrupted CSF flow and craniocervical instability [62]. Approximately 70–80% of intracranial fluid flows through the venous system, with around 70% of blood exiting the brain through the IJVs in the supine position [104]. Compression of the IJVs in the supine position is an established cause of elevated intracranial pressure, which was seen in this patient cohort, suggesting it may be a missing underlying cause of many hEDS symptoms [105].

Transorbital ultrasonographic examination of the ONSD as used in this study is a reliable, noninvasive diagnostic tool employed in many settings to detect elevated intracranial pressure, which is shown to correlate with invasive and noninvasive measurements of intracranial pressure [106,107,108]. Increased ONSD is indicative of elevated intracranial pressure because it provides a picture of increased CSF accumulated around the optic nerve through the subarachnoid space. The mean ONSDs in this cohort were 7.28 mm and 7.17 mm on the left and right, respectively. The established normal unilateral ONSD values in healthy adults range from <5.0–5.4 mm [109]. When evaluating for elevated intracranial pressure, the widely accepted cutoff value used in emergency rooms and other settings ranges from >5.6–6.0 mm, although some have found as low as >5.3–5.7 mm to be correlated with elevated intracranial pressure > 20 mmHg (normal is 10–15 mmHg) [108,110,111,112]. This finding of elevated ONSD supports the idea that increased intracranial pressure contributes to the clinical characteristics of the patients in this cohort [113]. Similar findings of IJV compression at C1 and an increase in ONSD were found in a separate cohort of patients with symptoms of anxiety, brain fog, concentration difficulty, depression, headaches, obsessive thoughts, panic attacks, and rumination [32,114].

**Figure 14:**
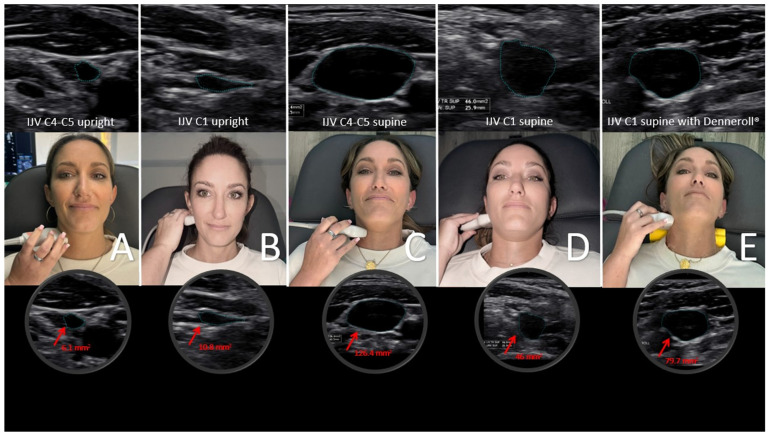
Internal jugular vein (IJV) cross-sectional area (CSA) measurement using ultrasound in various head and neck positions. (**A**) Upright measurement at the C4–C5 level. (**B**) Upright measurement at the atlas (C1) level. (**C**) Supine measurements at the C4–C5 level while lying on the Denneroll^®^. (**D**) Supine measurements at the C1 level. (**E**) Supine measurements at the C1 level while lying on the Denneroll^®^. The internal jugular vein is typically much more open in the supine position compared to upright, and when there is a breakdown of the cervical curve (dysstructure) and/or ligamentous cervical instability, the IJV gets maximally compressed at the atlas. Figure copyright belongs to authors. (Reproduced from Ref. [29]).

The internal jugular veins are easily, noninvasively, and reliably measured using B-mode ultrasound, as utilized in this study [115] (Figure 14). Normal IJV CSA in the supine position is >90–100 mm^2^ and approximately 25 mm^2^ in the upright position [44,116]. These data show that the cross-sectional area using ultrasound of the IJVs in the mid-neck (C4–C5) and upper neck (C1–C2) in both a seated and supine position and with different head and neck positions can determine not only if the IJVs are being compressed, but by how much and which positions and locations are worse.

**Figure 15:**
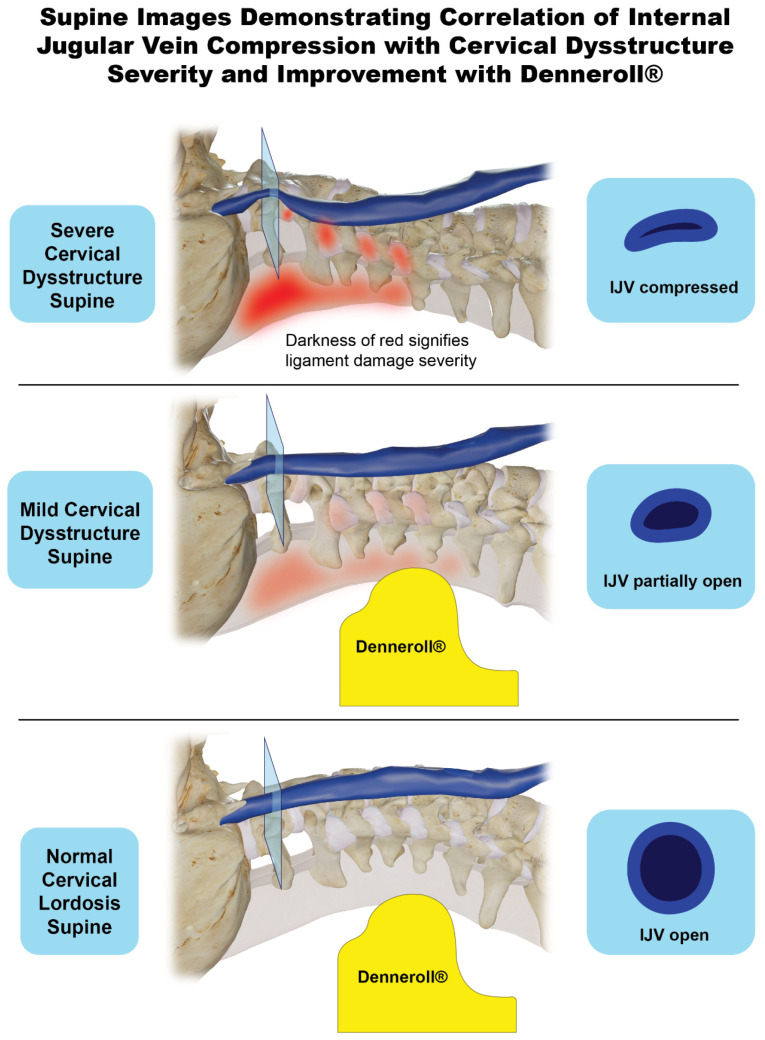
Supine images demonstrating correlation of internal jugular vein compression with cervical dysstructure severity and improvement with Denneroll^®^. Figure copyright belongs to authors.

This cohort demonstrated significant improvement in IJV CSA at C1 when lying on a cervical orthotic device, the Denneroll^®^, compared to supine without it (105.1 mm^2^ vs. 80.4 mm^2^, *p* < 0.001). By lying on the Denneroll^®^, a cervical extension traction device, the cervical curve is encouraged to go into lordosis, a documented type of therapy primarily used in chiropractic treatment to improve cervical lordosis and relieve symptoms from forward head posture, such as chronic pain [117,118,119] (Figure 15). The increased IJV CSAs seen in this study when lying on the cervical orthotic device support the notion that cervical structural changes are a cause of venous obstruction, which may be corrected by restoring the cervical lordotic curve. We further hypothesize that by following a curve correction program and restoring structural integrity of the cervical spine to maintain proper lordosis, the IJV compression by the atlas should be relieved, potentially accompanied by a reduction in symptoms.

Cerebral venous outflow obstruction is well established to coexist with connective tissue disorders and can account for many symptoms such as headache, dizziness, tinnitus, and cognitive dysfunction [120]. Cervical IJV compression at the C1 transverse process (also identified in the current literature as between the C1 transverse process and the styloid process) is associated with many clinical manifestations, including such nonspecific neurological symptoms as neck pain, headache, visual disturbances, headaches, dizziness, and mental health conditions, though the etiology is often unspecified [96,121]. In a study of 108 consecutive patients undergoing CT angiography for *presumed arterial obstruction*, 50% of them had IJV compression, and 93% of the compressions were at the level of C1 [122]. Cerebral venous outflow obstructions, including those in the IJVs, are also increasingly recognized in cases of intracranial hypertension that were previously identified as “idiopathic.” [123,124] Many symptoms from this condition, including headache, tinnitus, dizziness, and cognitive dysfunction, improve dramatically with restoration of IJV and cerebral venous outflow [125,126,127]. Intracranial pressure reduces, and related symptoms such as headache, tinnitus, and optic papilledema may resolve following IJV stenting or styloidectomy, the main surgical treatments for internal jugular vein stenosis/styloid-jugular venous compression syndrome, though the long-term outcomes are unknown, highlighting the importance of further investigating underlying causes in order to advance innovative treatments [127,128,129]. In a study involving lumbar puncture positioning, neck flexion produced an intracranial pressure 15.2 mmHg higher than when straightening the neck in the same seated position, evidence that cervical structural position influences brain pressure [130].

**Figure 16:**
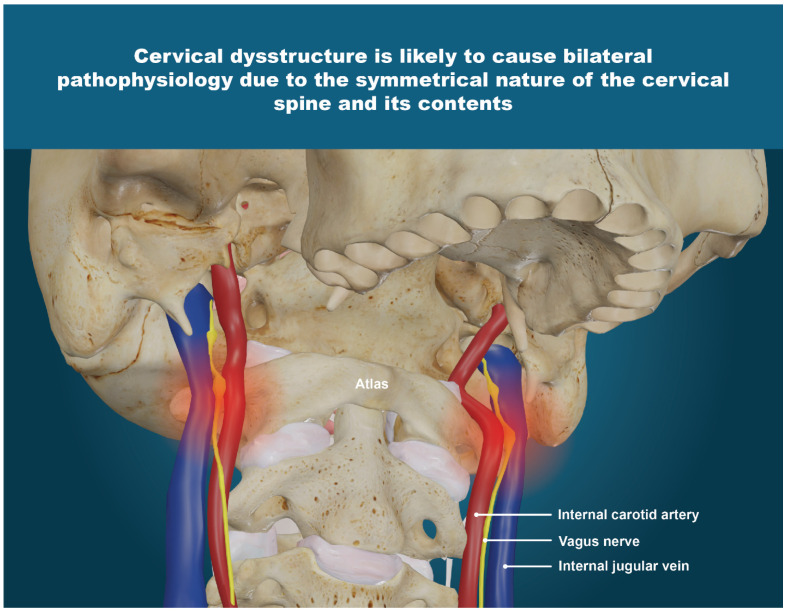
Cervical dysstructure is likely to cause bilateral pathophysiology due to the symmetrical nature of the cervical spine and its contents. Figure copyright belongs to authors.

Tonometry, or intraocular pressure, was included in this study because of its often-direct relationship to intracranial pressure; for instance, both are increased by IJV compression [131,132]. In about two-thirds of cases, a rise in intraocular pressure is associated with a rise in intracranial pressure [133]. In this study, the average bilateral total intraocular pressure was 38.34 mmHg (normal being < 42.0 mmHg), with no significant differences seen between left and right eyes, while 34.5% of patients had ocular hypertension (intraocular pressure > 42.0 mmHg). While the actual measurements of tonometry, pupillometry, and IJV and vagus nerve CSA are done individually (right, then left), they are reported and discussed here as bilateral totals to streamline comparisons, since there was no statistically significant difference found between right- and left-side measurements. We postulate that the reason for the bilateral findings is due to the concurrent findings of ligamentous cervical instability, forward head posture, and loss of cervical lordosis (Figure 16). This is the first study to document not only IJV CSA, but also ONSD and intraocular pressure in patients with hEDS and chronic symptoms. If validated by further prospective studies, the identification of IJV compression as an underlying contributor to the clinical characteristics of hEDS patients would be of significant clinical relevance, as it would highlight a potentially modifiable pathology with implications for improved patient outcomes.

### 4.3. Vagus Nerve Involvement in hEDS Patients

This is the first time ultrasound vagus nerve CSA has been reported in a cohort of patients with hEDS, a remarkably novel finding as 100% of the patients in this study had evidence of bilateral vagus nerve degeneration (atrophy). Normal cross-sectional areas of the vagus nerves in healthy adults average between 1.9 and 2.5 mm^2^, the right often larger than the left [37,42]. This patient population presented with significantly decreased vagus nerve CSA, with averages of 1.22 mm^2^ on the right and 1.34 mm^2^ on the left and a total bilateral average of 2.55 mm^2^. The vagus nerves are very susceptible to compression and degeneration, which can be documented by ultrasound of the carotid sheath [134,135]. A decrease in vagus nerve CSA is found to correlate with parasympathetic dysfunction, which may explain many symptoms present throughout this patient population [41]. The vagus nerves are the dominant nerves of parasympathetic nervous system, accounting for 80–90% of its afferent fibers, and are the primary inhibitor of sympathetic stimulation [136]. Degeneration or dysfunction of the vagus nerves, resulting in reduced vagal activity, could then lead to a sympathetic-dominant state. Hyperactivity of the sympathetic system and/or parasympathetic hypoactivity causes sympathovagal imbalance, something seen in a host of brain and body diseases, including mental health disorders [136,137,138]. Damage to the vagus nerves, or decreased vagal signaling, results in impaired anti-inflammatory effects or excessive production of inframammary cytokines [139]. The potential sympathetic dominance and excessive systemic inflammation may contribute to many of hEDS patients’ constellation of symptoms, including joint pain, gastrointestinal disturbances, fatigue, and more, due to the vagus nerves’ role in the brain–body connection and in regulating inflammation [140,141].

The mystery of the symptoms of hEDS often leads to the diagnosis of functional neurologic or gastrointestinal disorders [142]. Vagus nerve dysfunction can affect nearly every organ in the body, with consequences including abnormal heart rate and blood pressure, digestive issues, loss of respiratory control, and chronic pain, which may explain the high correlation between joint hypermobility and functional neurologic disorders [143,144]. In the latter, the symptoms are real, but there is not an identified structural problem or organ damage; it is thought to be a brain function or network communication problem, which could be from overlooked dysfunctional vagus nerves [145]. While the term “functional” often denotes psychological or unbeknownst neurologic dysfunction, our study could offer an explanation that there is a structural vagopathy.

The decrease in vagus nerve CSA can be explained in our cohort by the dynamic carotid sheath compression, which occurred primarily at the atlas, the very position where the two vagus nerve ganglia reside. The superior (jugular ganglion) is located in the jugular foramen and the inferior (nodose ganglion) sits right in front of the atlas. The nodose ganglion carries crucial sensory information from the internal organs, including the heart, lungs, and digestive tract [144].

Research shows that up to 98% of hEDS/hypermobile spectrum disorder subjects had functional gastrointestinal disorders, compared to 47% of controls [146]. Functional neurological symptoms are present in up to 92% of hEDS patients [142,147]. Dynamic carotid sheath compression, which can occur when the neck becomes chronically flexed, puts the contents of the carotid sheath, as well as other neurovascular structures, under constant stretch and tension. It could be one reason why many hEDS patients are diagnosed with functional disorders, such as functional internal organ or neurological disorders, meaning the symptoms are severe but they are not caused by a recognizable organ disease or injury [142].

Up to two-thirds of EDS patients have evidence of small fiber neuropathy [148]. Small-fiber dysfunction has been indicated to worsen dysautonomia and vascular maladaptation in hEDS [149]. Symptoms such as weakness, fatigue, seizures, and sensory disorders documented in so-called “functional neurologic disorders” where no structural abnormalities are identified in scans such as brain, brainstem, and spinal cord MRIs, could be explained by dynamic carotid sheath compression from ligament laxity [150]. In functional neurologic disorders, the condition remains largely unexplained, but the nervous system does not function properly, leading to symptoms such as weakness, fatigues, seizures, and sensory issues [142,150,151]. Some studies have found exaggerated stress reaction (somatosensory amplification, or brain hypersensitivity syndrome) in patients with hypermobility syndromes, which we propose could have underlying cerebral neuropathology from IJV compression and resultant increased intracranial pressure or vagus nerve degeneration [152,153,154].

**Figure 17:**
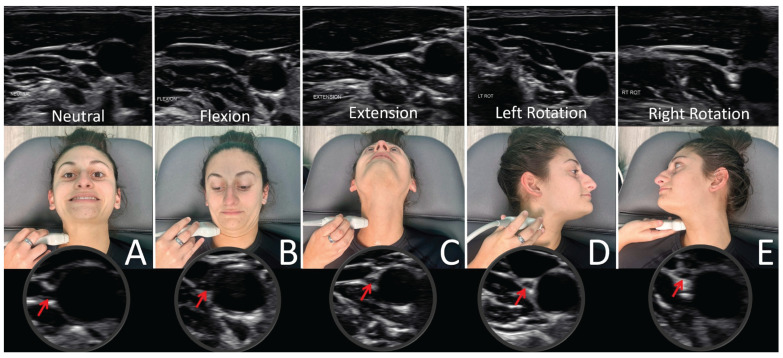
Ultrasound of vagus nerve (red arrows) in mid-cervical region with various neck positions. (**A**) Neck in neutral position. (**B**) Flexed neck. (**C**) Neck extended. (**D**) Neck rotated left. (**E**) Neck rotated right. As can be seen, the vagus nerve within the carotid sheath undergoes various structural tensions depending on neck positions. Figure copyright belongs to authors. (Reproduced from Ref. [29]).

Dysautonomia and autonomic nervous system dysfunction are frequent extraarticular manifestations of hEDS [61,155]. Some 75% percent of our cohort was diagnosed with dysautonomia; the prevalence in hEDS ranges from 31 to 94% [17]. Dysautonomia or autonomic nervous system dysfunction can have many different etiologies, including those involving cardiac, endocrine and immune systems, brainstem and spinal reflexes, fluid status, and many others [156]. Patients with hEDS and dysautonomia were found by standard autonomic nervous system tests such as heart rate variability (frequency domain) to have a higher LF/HF ratio at rest, a sign of increased sympathetic/parasympathetic activity compared to controls [66]. The vagus nerves traverse the neck just anterior to the cervical vertebrae, as do the IJVs, and are subject to injury from stretch and compression, as all nerves are [157]. Vagus nerve CSA can be seen using ultrasound, as utilized in this study. (Figure 17). Based on the findings of vagus nerve degeneration and cervical structural abnormalities in this cohort, we propose an additional potential cause of dysautonomia to be cervicovagopathy—vagus nerve degeneration secondary to cervical pathology [158].

Patients with dysautonomia are known to have a plethora of symptoms involving the cardiovascular, gastrointestinal, neurological, cognitive, and other body systems, with hEDS and other hypermobility syndromes being a common co-morbidity [159,160]. In a different cohort of patients with anxiety, dizziness, fatigue, irritability, lightheadedness, insomnia, sleeping difficulty, neck pain, and neck cracking and grinding, vagus nerve atrophy (degeneration) was also overwhelmingly present [158]. Vagus nerve degeneration has been found in progressive neurodegenerative disorders, and vagus nerve stimulation has been used for treatment of those disorders, as well as for symptoms such as anxiety, depression, and headache [161,162,163,164]. Although reduced vagus nerve CSA was observed, functional autonomic testing was not available in this retrospective dataset. The relationship between structural findings and vagal function in this cohort cannot therefore be determined. Future prospective studies incorporating standardized autonomic assessments are needed to evaluate potential functional implications in hEDS.

The pupillometry findings observed in this study may be suggestive of a sympathetic dominant state, with pupil diameter bilateral totals averaging 10.83 mm (normal is <8 mm) and bilateral total percent constriction to light averaging 74.9% (normal is 30–60%), both in the “high end” of normal or slightly above normal [165,166]. Pupillometry has been used to document such conditions as acute rises in intracranial pressure, as also occurs after trauma or in intensive care units following brain surgery. It is also a non-specific indicator of sympathetic hyperactivity [167,168,169]. While these findings alone do not confirm sympathetic dominance, pupillometry may be included in future studies involving similar patient cohorts, where symptoms suggestive of sympathetic dominance are prevalent, to further assess clinical relevance.

**Figure 18:**
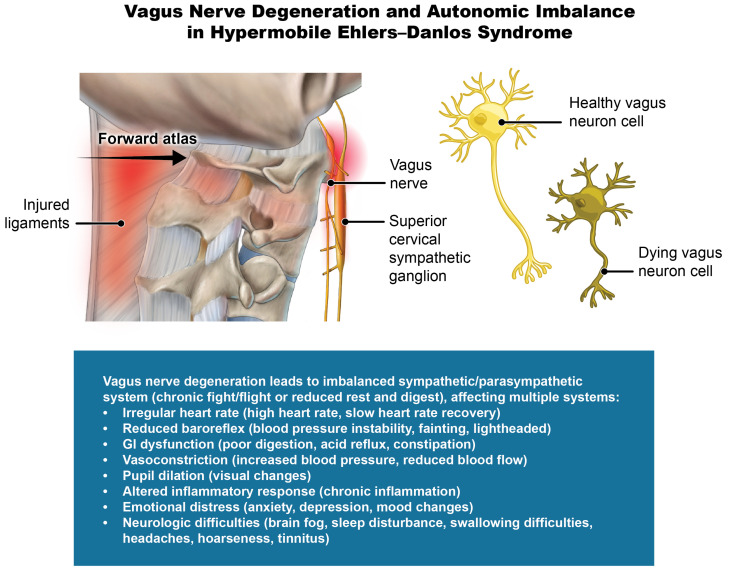
Vagus nerve degeneration and autonomic imbalance in hypermobile Ehlers–Danlos syndrome. Figure copyright belongs to authors.

As we previously described, when vagus nerve dysfunction is caused by structural neck (cervical spine) issues, it is called “cervicovagopathy” [158]. We suspect many symptoms in this patient population with hEDS could be caused by vagus nerve degeneration, and therefore autonomic dysfunction. (Figure 18). Validation of vagus nerve degeneration as an underlying factor would not only clarify a pathophysiological basis for the frequent presentation of dysautonomia and myriad symptoms seen in hEDS, but it would also serve as a target for intervention that could improve clinical outcomes. If vagus nerve degeneration is in fact due to cervical stretch and compression from a cervical spine pathology, then a multifaceted treatment program may include ergonomics, postural exercises, a vagus nerve stimulator, and prolotherapy, with an overall aim of correcting the underling cervical dysstructure (such as restoring cervical lordosis and stabilizing identified ligamentous cervical spine instabilities), thereby reducing stretch and compression of the nerves [158].

### 4.4. Clinical Relevance and Future Directions

While previous studies have described joint hypermobility/instability and cervical spine instability associated with complications in hEDS patients, as well as autonomic, cerebrovascular, and neuropathic features of hEDS, consideration of the structural mechanistic framework for the widespread multisystem presentation of the condition has been limited [11,18,25,79,94,142,147,170]. This study builds on the current literature and aligns with prior observations from specialists managing this challenging and poorly understood patient population, suggesting that upper cervical insatiably may be an important contributor to the complex clinical profile in a subset of the hEDS patients [71,79,85,171].

The findings offer novel evidence that cervical spine abnormalities could contribute to the understanding of multisystem involvement in hEDS, including involvement of the autonomic nervous system via the vagus nerves and of cerebral venous outflow obstruction via the IJVs. Given that patients with hEDS are inherently prone to upper cervical spine morphology, these findings may help elucidate the consequences of that vulnerability. It is plausible to consider how the structural cervical spine changes lead to a forward-shifted atlas encroaching on the carotid sheath, causing neurologic and vascular compression, substantiated by the documented ligamentous cervical instability, forward head posture, and loss of cervical lordosis in this cohort.

By highlighting previously underrecognized potential mechanisms, these findings could help address an existing gap in the treatment of hEDS, especially in the clinical context of otherwise elusive symptoms and complex cases. Ultimately, we propose that correction and stabilization of the cervical spine structure in patients with hEDS could help relieve symptoms stemming from ligament laxity/ligamentous instability in the cervical spine and relieve the potentially consequential IJV compression and vagus nerve degeneration. Prolotherapy is considered a regenerative injection technique that initiates the body’s natural three-phase healing process of inflammation, proliferation, and remodeling to create a controlled inflammatory response that encourages tissue repair in areas with poor blood supply, such as ligaments, tendons, and cartilage [172,173]. Prolotherapy has been shown to increase ligament strength and has been used successfully in cervical conditions, including instability [174,175,176,177,178].

This study highlights the need for larger prospective studies on underlying cervical spine pathology and its pathophysiological effects in patients diagnosed with hEDS, as well as potential improvement following cervical spine corrective treatment. The testing methods described in this study are objective, noninvasive, and reproducible. They can be done serially to check patient progress. Structural–neurovascular relationships similar to those observed in this hEDS cohort may warrant investigation in other conditions involving cervical instability, degenerative changes, or altered biomechanics, such as acquired ligament laxity and cervical spondylosis. Hypothetically, if cervical neck structure, including ligamentous cervical instability and cervical dysstructure, are the etiology of some widespread symptoms, then improving cervical lordosis and ligamentous stability should decrease or resolve them. If future studies demonstrate that improvements in neck structure, such as restoring cervical lordosis and improving stability, lead to IJV and vagus nerve CSAs’ improvement that corresponds with symptom improvement, then a great step forward in the alleviation of chronic and disabling hEDS symptoms would occur. We emphasize these data to be hypothesis-generating and that comparative, prospective studies will be necessary to determine the presence, magnitude, and clinical relevance of these observed structural and neurovascular associations.

## 5. Limitations

This study was designed as a descriptive, cross-sectional characterization of initial clinical test findings. Given the retrospective nature of the study, the findings cannot determine causation; they only describe associations. No inferential subgroup analyses were pre-specified. Although symptom presentation was clinically heterogeneous, the primary aim was to describe overall patterns of structural and neurovascular measurements within this cohort for consideration of potential underlying mechanisms contributing to hEDS, rather than to evaluate symptom-specific associations. Future studies with larger samples may be better positioned to examine subgroup differences.

Formal autonomic testing was not systematically collected as part of routine clinical care to assess vagus nerve function and were therefore not available for analysis in this dataset. As such, we are unable to directly correlate vagus nerve cross-sectional area with functional measures. We did not examine what led to the forward head posture, loss of lordosis, or LCI, but one could speculate that the modern lifestyle that includes excessive time spent in poor posture using computers and cell phones could contribute, compounded by the fact that patients with hEDS are predisposed to ligament laxity [179]. As data were collected from a single outpatient neck center, the findings may not be fully generalized to all clinical populations, though they do provide insight into a subset of patients with chronic, disabling symptoms who present with hEDS in the absence of other known mechanisms, highlighting interdisciplinary clinical relevance. Imaging interpretation was not performed under blinded research conditions. Although the imaging modalities used have established reliability in prior studies, the absence of formal blinding may introduce observer bias. Since this study is a retrospective chart review conducted at a single institution, inter-rater reliability was not formally calculated, although standardized data collection protocols were used.

The lack of an internal control group and matched comparisons limits causal interpretation of the observed associations. While we did exclude patients with previous neck injury, surgery, or trauma, potential confounding variables (such as medications, prior interventions, and lifestyle) were not fully stratified. Further, the absence of detailed genetic profiling limits the ability to exclude potential coexisting genetic factors that may contribute to the observed clinical features. This study does not include follow-up data to evaluate symptom progression or response to treatment. Future prospective studies with appropriate controls and predefined stratification strategies will be necessary to determine the specificity and mechanistic relevance of these findings. Further longitudinal studies are necessary to explore potential treatment options such as physical therapy, chiropractic adjustments, cervical curve correction, and prolotherapy, targeting restoration and stability of the cervical curve to relieve the pressure on vital neurovascular structures (IJV and vagus nerve) and evaluate efficacy.

## 6. Conclusions

This retrospective study is the first to quantify and document ligamentous facet joint cervical instability at C1–C2, evidence of IJV compression at C1, and vagus nerve degeneration in a cohort of patients with hEDS. Radiologically identified loss of cervical lordosis and forward head posture were also ubiquitous. More than 70% of patients reported more than 29 symptoms, emphasizing the systemic effects of the condition. The consistency of the cervical spine pathology and neurovascular abnormalities throughout the entire cohort supports potential cervical spine involvement in hEDS presentation. Recognition of cervical spine pathology in patients with hEDS may present patients with new targeted treatment options. Future prospective studies are needed to validate these associations and clarify underlying mechanisms contributing to the chronic symptomatology of hEDS, which could inform diagnosis, treatment, or preventative strategies. If cervical spine pathology proves to cause contributing mechanisms leading to complications, then nonsurgical correction of the cervical curve and cervical spine stabilization should be considered as part of a multidisciplinary approach to patients with hEDS.

## Figures and Tables

**Table 1:** Structural diagnostic imaging methods. Table copyright belongs to authors.

Measurement	Modality and Output	Criteria for Interpretation	Application
Ligamentous Upper Cervical Instability (C1–C2) Lateral Flexion	Upright digital motion X-ray (DMX, videofluoroscopy) of the cervical spine is used for identifying translation of adjacent vertebra (mm).	Normal is <2 mm in any direction	DMX allows structural deviations to be seen *during movements* and amongst different positions, which can document vertebral translations that could be putting strain on vital structures in the area. The upright position provides a high degree of accuracy which may otherwise be missed.
Ligamentous Cervical Instability (C2–C6) Flexion and Extension			
Depth of Curve	Upright cone beam CT of the cervical spine is used to identify distance from the posteroinferior aspect of the C4 vertebra to a line drawn from the posteroinferior aspect of the C6 vertebral body to the peak of the dens of C2 (mm).	Normal is 7–17 mm	Depth of curve is used to objectively assess the state of cervical lordosis.
C6-Atlas Interval (C6AI)	Upright cone beam CT of the cervical spine used to identify horizontal distance between the posterior border of the C6 vertebral body and a line drawn perpendicular from the anterior arch of the atlas in the sagittal view (mm).	Normal is <10 mm	C6AI objectively assesses the structural relationship of the atlas in relation to the lower cervical spine (C6) in the sagittal plane, providing an objective measurement for “forward head posture” as it identifies to the position of the head, which sits upon the atlas, compared to the lower neck.

**Table 2:** Neck vitals analysis methods. Table copyright belongs to authors.

Measurement	Modality and Output	Criteria for Interpretation	Notes/Application
Pupil Diameters	NeurOptics NPi^®^-200 pupillometer automatically measures baseline pupil diameter (mm).	Normal is 2–4 mm unilateral in lit environments	Chronically dilated pupils (mydriasis) can indicate sympathetic nervous system dominance/parasympathetic dysfunction (state of stress on the body).
Pupillary Light Reflex (Percent Change)	NeurOptics NPi^®^-200 pupillometer automatically calculates the percent change in the pupil diameter after initiating a flash of light (%).	Normal is 15–30% unilateral	Pupillary light reflex is used in clinical settings to assess for dysautonomia. Excessive percent change can be an indicator of sympathetic nervous system dominance.
Internal Jugular Vein Cross-sectional Area (IJV CSA)	Canon Aplio a550 ultrasound with 7 MHz linear probe is used to identify cross-sectional areas in multiple positions including seated, supine, and with use of cervical orthotic (mm^2^).	Normal is 90–100 mm^2^ unilateral	Abnormally small IJV CSA is a cause of venous outflow obstruction, and indicative of external compression.
Vagus Nerve Cross-sectional Area (Vagus nerve CSA)		Normal is >2.1 mm^2^ unilateral	Atypical vagus nerve CSA is indicative of degeneration and low vagal tone.
Optic Nerve Sheath Diameter (ONSD)	Canon ultrasound, ocular setting is used to identify the optic nerve sheath diameter (mm).	Normal is <6.1 mm unilateral	Elevated optic nerve sheath diameters can be indicative of increased cerebral spinal fluid and elevated intracranial pressure.
Intraocular Pressure (IOP)	iCare ic200 tonometer automatically measures intraocular pressure (mmHg).	Normal is <21 mmHg unilateral	Elevated intraocular pressure can be indicative fluid outflow obstruction and/or elevated venous pressure.

**Table 3:** Demographics and symptoms frequency at initial intake of 84 patients with hEDS at an outpatient neck center. Table copyright belongs to authors.

Demographics	Count	Percentage
Female	71	85%
Male	13	15%
Average age	35	-
**Number of reported symptoms at initial intake from hEDS patients at an outpatient neck clinic (n = 84).**		
**Number of Symptoms**	**Count of Patients**	**Percentage of Patients**
0–9	2	2.4%
10–19	12	14.3%
20+	70	83.3%
**Symptom**	**Count of Patients**	**Percentage of Patients**
Brain fog	80	95.2%
Fatigue	79	94%
Headaches	78	92.9%
Concentration difficulty	77	91.7%
Neck pain	77	91.7%
Anxiety	75	89.3%
Lightheadedness	75	89.3%
Dizziness	74	88.1%
Sleeping problems	73	86.9%
Muscles spasms/tension	72	85.7%
Neck grinding/cracking	70	83.3%
Sensitivity to light	70	83.3%
Digestion problems	69	82.1%
Irritability	68	81.0%
Nausea	67	79.8%
Stiffness in joints	67	79.8%
Balance difficulties	66	78.6%
Bruises easily	65	77.4%
Constipation	65	77.4%
Ringing in ears	65	77.4%
Insomnia	64	76.2%
Sensitivity to sound	64	76.2%
Blurred vision	63	75.0%
Dysautonomia	63	75.0%
Ear fullness/pressure	63	75.0%
Weakness	63	75.0%
Depression/hopelessness	61	72.6%
Eye pain/pressure	61	72.6%
Heart palpitations	60	71.4%

**Table 4:** Frequency of abnormal objective cervical structural and neck vitals test results in 84 patients with hEDS at an outpatient neck center. Table copyright belongs to authors.

Method	N	Normal Range Cutoff	% Abnormal
C6-atlas interval *	82	<10 mm	98.8%
Depth of curve **	79	7–10 mm	94.9%
Ligamentous cervical instability (LCI) Extension	82	<4 mm	52.4%
LCI Flexion	83	<4 mm	55.4%
Internal jugular vein (IJV) Denneroll^®^ C1 total	80	>180 mm	90%
IJV supine C1 left	84	>90 mm	91.7%
IJV supine C1 right	84	>90 mm	95.2%
IJV supine C1 total	84	>180 mm	96.4%
IJV supine C4–C5 left	84	>90 mm	72.6%
IJV supine C4–C5 right	84	>90 mm	69%
IJV supine C4–C5 total	84	>90 mm	72.6%
Intraocular pressure left	84	<21 mmHg	34.5%
Intraocular pressure right	84	<21 mmHg	30.9%
Intraocular pressure total	84	<42 mmHg	34.5%
LCI C1–C2 lateral flexion left	84	<2 mm	89.3%
LCI C1–C2 lateral flexion right	84	<2 mm	88.1%
LCI C1–C2 lateral flexion total	84	<4 mm	90.2%
Optic nerve sheath diameter left	83	<6.1 mm	87.9%
Optic nerve sheath diameter right	83	<6.1 mm	80.7%
Optic nerve sheath diameter total	83	<12.2 mm	92.8%
Percent change total	84	30–60%	92.9%
Pupil diameter total	84	<8 mm	95.2%
Styloid length total	83	<60 mm	19.2%
Vagus nerve CSA left	84	>1.9 mm	91.7%
Vagus nerve CSA right	84	>2.1 mm	98.8%
Vagus nerve CSA total	84	>4.2 mm	100%

* Depth of Curve = horizontal distance in the sagittal plane from posterior inferior C4 vertebra to line drawn from posterior inferior C6 vertebra to top of dens (optimal is 7–17 mm). ** C6-Atlas Interval = C6AI = horizontal distance in the sagittal plane of the posterior inferior C6 vertebra to anterior atlas (optimal is <10 mm).

**Table 5:** Mean and standard deviation objective cervical structural and neck vitals test results in 84 hEDS patients at an outpatient neck clinic. Table copyright belongs to authors.

	N	Mean	SD
C6-atlas interval (mm)	82	39.12 mm	12.18
Depth of curve (mm)	79	1.58 mm	3.24
Ligamentous cervical instability (LCI) Extension (mm)	82	4.41 mm	3.22
LCI Flexion (mm)	83	5.29 mm	3.38
IJV Denneroll^®^ C1 left (mm^2^)	80	49.35 mm^2^	27.72
IJV Denneroll^®^ C1 right (mm^2^)	80	55.73 mm^2^	30.84
IJV Denneroll^®^ C1 total (mm^2^)	80	105.08 mm^2^	43.51
IJV seated C1 total (mm^2^)	84	19.55 mm^2^	11.74
IJV seated C4–C5 total (mm^2^)	84	21.00 mm^2^	14.84
IJV supine C1 left (mm^2^)	84	40.97 mm^2^	29.43
IJV supine C1 right (mm^2^)	84	39.94 mm^2^	25.03
IJV supine C1 total (mm^2^)	84	80.4 mm^2^	42.26
IJV supine C4–C5 left (mm^2^)	84	67.91 mm^2^	42.53
IJV supine C4–C5 right (mm^2^)	84	71.48 mm^2^	45.36
IJV supine C4–C5 total (mm^2^)	84	139.21 mm^2^	72.59
Intraocular pressure total (mmHg)	84	38.34 mmHg	8.31
Intraocular pressure left (mmHg)	84	19.14 mmHg	4.15
Intraocular pressure right (mmHg)	84	19.08 mmHg	4.15
LCI C1–C2 lateral flexion total (mm)	83	7.84 mm	3.18
Percent change/light constriction total (%)	84	74.92%	8.93
Percent change/light constriction left (%)	84	37.7%	6.05
Percent change/light constriction right (%)	84	36.8%	5.02
Optic nerve sheath diameter total (mm)	83	14.45 mm	1.64
Optic nerve sheath diameter left (mm)	83	7.28 mm	0.91
Optic nerve sheath diameter right (mm)	83	7.17 mm	1.00
Pupil diameter total (mm)	84	10.83 mm	1.79
Pupil diameter right (mm)	84	5.32 mm	0.86
Pupil diameter left (mm)	84	5.51 mm	0.99
Styloid length total (mm)	83	43.14 mm	28.35
Vagus nerve CSA total (mm^2^)	84	2.55 mm^2^	0.59
Vagus nerve CSA left (mm^2^)	84	1.34 mm^2^	0.38
Vagus nerve CSA right (mm^2^)	84	1.22 mm^2^	0.34

## Data Availability

The original contributions presented in this study are included in the article. Further inquiries can be directed to the corresponding author.
